# Longitudinal Brain Magnetic Resonance Imaging CO_2_ Stress Testing in Individual Adolescent Sports-Related Concussion Patients: A Pilot Study

**DOI:** 10.3389/fneur.2016.00107

**Published:** 2016-07-08

**Authors:** W. Alan C. Mutch, Michael J. Ellis, Lawrence N. Ryner, Marc P. Morissette, Philip J. Pries, Brenden Dufault, Marco Essig, David J. Mikulis, James Duffin, Joseph A. Fisher

**Affiliations:** ^1^Department of Anesthesia and Perioperative Medicine, University of Manitoba, Winnipeg, MB, Canada; ^2^University of Manitoba, Winnipeg, MB, Canada; ^3^Health Sciences Centre, Winnipeg, MB, Canada; ^4^Canada North Concussion Network, Winnipeg, MB, Canada; ^5^Department of Surgery, University of Manitoba, Winnipeg, MB, Canada; ^6^Department of Pediatrics and Child Health, University of Manitoba, Winnipeg, MB, Canada; ^7^Section of Neurosurgery, University of Manitoba, Winnipeg, MB, Canada; ^8^Pan Am Concussion Program, Winnipeg, MB, Canada; ^9^Children’s Hospital Research Institute of Manitoba, University of Manitoba, Winnipeg, MB, Canada; ^10^Pan Am Clinic Foundation, Winnipeg, MB, Canada; ^11^Department of Radiology, University of Manitoba, Winnipeg, MB, Canada; ^12^College of Medicine, University of Manitoba, Winnipeg, MB, Canada; ^13^Department of Community Health Sciences, University of Manitoba, Winnipeg, MB, Canada; ^14^Department of Medical Imaging, University of Toronto, Toronto, ON, Canada; ^15^University Health Network Cerebrovascular Reactivity Research Group, Toronto, ON, Canada; ^16^Department of Physiology, University of Toronto, Toronto, ON, Canada; ^17^University of Toronto, Toronto, ON, Canada; ^18^Department of Anesthesia, University of Toronto, Toronto, ON, Canada

**Keywords:** sports-related concussion, adolescent, post-concussion syndrome, magnetic resonance imaging, blood oxygen level-dependent imaging, cerebrovascular reactivity, longitudinal

## Abstract

**Background:**

Advanced neuroimaging studies in concussion have been limited to detecting group differences between concussion patients and healthy controls. In this small pilot study, we used brain magnetic resonance imaging (MRI) CO_2_ stress testing to longitudinally assess cerebrovascular responsiveness (CVR) in individual sports-related concussion (SRC) patients.

**Methods:**

Six SRC patients (three males and three females; mean age = 15.7, range = 15–17 years) underwent longitudinal brain MRI CO_2_ stress testing using blood oxygen level-dependent (BOLD) MRI and model-based prospective end-tidal CO_2_ targeting under isoxic conditions. First-level and second-level comparisons were undertaken using statistical parametric mapping (SPM) to score the scans and compare them to an atlas of 24 healthy control subjects.

**Results:**

All tests were well tolerated and without any serious adverse events. Anatomical MRI was normal in all study participants. The CO_2_ stimulus was consistent between the SRC patients and control subjects and within SRC patients across the longitudinal study. Individual SRC patients demonstrated both quantitative and qualitative patient-specific alterations in CVR (*p* < 0.005) that correlated strongly with clinical findings, and that persisted beyond clinical recovery.

**Conclusion:**

Standardized brain MRI CO_2_ stress testing is capable of providing a longitudinal assessment of CVR in individual SRC patients. Consequently, larger prospective studies are needed to examine the utility of brain MRI CO_2_ stress testing as a clinical tool to help guide the evaluation, classification, and longitudinal management of SRC patients.

## Introduction

Concussion is a form of traumatic brain injury (TBI) that represents an important public health concern, especially in children and adolescents participating in sports. Although the vast majority will recover within 1–4 weeks of injury, an important proportion will develop prolonged symptoms or post-concussion syndrome (PCS) (1–6). This heterogeneous group of patients can be classified clinically into those with exercise intolerance, vestibulo-ocular dysfunction, cervical spine soft tissue injury, migraine headaches, and post-injury psychiatric disorders (7–12). In recent years, several advanced neuroimaging techniques have demonstrated significant differences in white matter anisotropy, brain activation patterns, and resting cerebral blood flow (CBF) between groups of concussion patients and healthy control subjects (13–23). Although these studies have advanced our understanding of the effects of concussion on brain structure and function, examination of group differences in neuroimaging biomarkers may not be an accurate reflection of the pathophysiological mechanisms underlying the symptoms and injury states in individual sports-related concussion (SRC) patients. Consequently, there remains a persistent need for neuroimaging assessment tools that can inform the evaluation and longitudinal management of individual concussion patients.

One of the metrics defining the health of the system responsible for regulating CBF during states of rest, activity, and disease is cerebrovascular reactivity/responsiveness (CVR), which is defined as the change in CBF in response to a vasodilatory stimulus (24, 25). Although previous studies have used techniques, such as transcranial Doppler ultrasonography to investigate changes in CVR following concussion (26–28), magnetic resonance imaging (MRI)-based assessment tools have several advantages, including superior spatial resolution and the ability to evaluate the entire brain (29–31). However, to provide accurate and longitudinal MRI-based assessment of CVR, a quantifiable, reliable, and reproducible vasoactive stimulus must be paired with an MRI sequence that can measure CBF or its surrogate (32, 33). Using model-based prospective targeting (MPET) of CO_2_ applied to blood oxygen level-dependent (BOLD) MRI, our group has introduced a brain MRI CO_2_ stress test that is capable of providing whole brain CVR mapping in individual concussion patients (34, 35). In a recent study, we demonstrated group and individual differences in CVR among adolescent PCS patients compared with healthy control subjects (35).

In this pilot study, we report results of longitudinal qualitative and quantitative brain MRI CO_2_ stress testing in individual adolescent SRC patients, comparing them to an atlas of healthy adolescent control subjects tested in the same way.

## Materials and Methods

### Research Design

This study was approved by the Biomedical Research Ethics Board at the University of Manitoba. We conducted a prospective case–control study of adolescent SRC patients and healthy control subjects. All adolescent SRC patients were recruited from the Pan Am Concussion Program in Winnipeg, MB, Canada. Patient inclusion criteria for this study included (1) diagnosis of SRC according to the definition set forth by the International Consensus on Concussion in Sport (36); (2) age 13–21 years. Healthy control subjects were recruited through word of mouth, including patient siblings and relatives. Control subject inclusion criteria included age 25 or younger. Control subject exclusion criteria were as follows: (1) the presence of a symptomatic concussion; (2) history of prior TBI or neurological condition resulting in structural brain abnormality on previous neuroimaging; (3) contra-indication to MRI; (4) presence of a neurological condition requiring prescription medication.

### Clinical Assessment

All adolescent SRC patients underwent a clinical evaluation by a single neurosurgeon. All healthy control subjects underwent clinical interview to collect demographic data, past medical history, and past concussion history. In general, patients were deemed completely recovered when they were asymptomatic, tolerating full-time school, and neurological examination results were normal. In-season athletes were also required to successfully complete the International Consensus on Concussion in Sport return-to-play guidelines (36) to be deemed completely recovered. In some cases, hybrid neuropsychological testing and graded aerobic treadmill testing were used to confirm clinical recovery as indicated by the treating neurosurgeon. Patients were diagnosed with physiological post-concussion disorder if they had symptoms for 1 month or longer and demonstrated a symptom-limited threshold on graded aerobic treadmill testing (8, 11). Patients were diagnosed with vestibulo-ocular post-concussion disorder if they demonstrated subjective and objective evidence of vestibulo-ocular dysfunction symptoms for 1 month or longer and had no clinical evidence of exercise intolerance (9). Patients were diagnosed with a post-injury psychiatric disorder if they developed a novel psychiatric disorder following a SRC (10).

### Neuroimaging Assessment

Following informed consent and clinical assessment, all subjects underwent neuroimaging assessment. SRC patients underwent initial neuroimaging assessment during the symptomatic phase of their injury and follow-up neuroimaging after documented clinical recovery. Follow-up neuroimaging assessment for SRC patients was scheduled based on patient and parent convenience rather than a pre-determined time point. MPET CO_2_ targeting was achieved by precise delivery of CO_2_ using a sequential gas delivery breathing circuit connected to a computerized gas-blender (RespirAct, Thornhill Research Inc., Toronto, ON, Canada). This device allows precise manipulation of PETCO_2_ levels under isoxic (target PETO_2_ = 115 mmHg) conditions. Hemodynamic monitoring during the study period included continuous heart rate and pulse oximetry and non-invasive blood pressure measurement (BP) at 3-min intervals.

All images were acquired using a Siemens Verio 3.0-T MR scanner with a 12-channel phased-array head coil. The MR-imaging protocol consisted of baseline anatomical imaging including sagittal 3D T1 MPRAGE (whole brain coverage; matrix: 256 × 256; slice thickness: 2.2 mm; no interslice gap; voxel size: 2 mm × 2 mm × 2 mm), axial fluid-attenuated inversion recovery (FLAIR), axial gradient recalled echo planar (GRE) sequences, and GRE B0-field mapping. CVR was assessed using continuous BOLD MRI during MPET CO_2_ targeting. The breathing sequence during BOLD imaging consisted of interval step-changes as follows: baseline ETCO_2_ (120 s), hypercapnia (5 mmHg above baseline for 120 s), baseline ETCO_2_ (30 s), hypercapnia (5 mmHg above baseline for 120 s), baseline ETCO_2_ (30 s), hypercapnia (5 mmHg above baseline for 120 s), baseline ETCO_2_ (120 s). BOLD MRI data were acquired with a T2*-weighted single-shot gradient echo pulse sequence with echoplanar readout (field of view: 24 cm × 24 cm; matrix: 64 × 64; TR: 2000 ms; TE: 30 ms; flip angle: 85°; slice thickness: 5.0 mm; interslice gap: 2.0 mm; voxel size: 3.75 mm × 3.75 mm × 6 mm; number of temporal frames = 330). Subject data were excluded if there was >3 mm of motion or inadequate end-tidal targeting.

#### Structural Neuroimaging

The structural neuroimaging component of each study was reviewed by a board-certified neuro-radiologist.

#### Preprocessing of MRI Sequences

Standard preprocessing of MRI EPI output was carried out for the BOLD sequences using SPM8 software and included batch processing by an SPM toolbox and custom written in-house MatLab scripts. The BOLD data were interpolated to the MPRAGE voxel dimensions. The fMRI model specification was a 2-pass process. We first modeled the data using the finite impulse response (FIR) package in SPM. The hypercapnic MPET model, as described earlier, using a triple stimulus block design for CO_2_ square wave response was assessed with a zero offset for this stimulus. The whole brain response to the CO_2_ stimulus was examined. An event-related response was calculated. The time to maximal response to hypercapnia was noted. This delay in response to the change in end-tidal CO_2_ was corrected based on a constructed series of time delay block stimulus files generated with delays from 0 to 30 s. This new fMRI model based on the time delay for brain activation by CO_2_ was now rerun as above.

We used the motion correction file generated with realignment as regressors in the model. First-level analysis results were based on this time-corrected analysis and the contrast images generated here were used in the second-level analysis. Masks were generated to correct for known inhomogeneous output of BOLD EPI signals. We made individualized masks based on GRE B0-field inhomogeneities greater than and less than 500 arbitrary units (AU). These binary images masked out voxels with inhomogeneities at the base of the brain, those adjacent to the petrous bone and those contiguous with the frontal sinuses. A dilated CSF mask with dilation × 2 (based on the Wake Forest University Pick Atlas) was generated to mask out the ventricular and periventricular inverse BOLD response in part related to choroid plexus response and interface changes at the ventricular edges (37). This combined binary image – labeled [(gm + wm) − (B0_inhomogeneity + dilated_ventricles)] was used in the second-level analysis as an inclusive mask to ensue that the abnormal voxels for an individual study were intraparenchymal. A representative mask is shown in the Supplementary Material. First-level analyses were undertaken with the author blinded to the subject’s group (healthy control versus SRC patient); however, second-level analyses were not blinded since they were based on the results of the first-level analysis.

### Statistical Analysis

#### Cerebrovascular BOLD Responsiveness (First-Level Analysis)

First-level analyses were undertaken for each study participant. BOLD increases in response to the hypercapnic breathing stimulus and BOLD decreases in response to the stimulus were assessed at the *p* = 0.001 uncorrected level. The cluster size threshold was 10 voxels. The response to the applied CO_2_ stimulus and its inverse were expressed as voxel counts/whole-brain voxel count ratios.

#### Cerebrovascular BOLD Responsiveness (Second-Level Analysis)

A second-level analysis for the BOLD studies was completed based on comparison to an atlas of adolescent control subjects (*n* = 24), thereby allowing individual longitudinal analysis of the six SRC patients. Each SRC patient study underwent voxel-by-voxel comparisons for BOLD signals that were less than or greater than the mean control group response. This second-level analysis was conducted over a series of *p*-values (*p* = 0.001, 0.005, 0.01, and 0.05; we report our results at the *p* = 0.005 level; see below). For each individual second-level comparison, the images were masked using the combined mask outlined above for that individual versus the atlas output, and the voxel counts reported are corrected for the applied individual mask.

## Results

### Participants

In this study, longitudinal brain MRI CO_2_ stress testing was carried out in six SRC patients (three males and three females; mean age = 15.7, range = 15–17 years) whose test results were compared with those of a normal control atlas of 24 subjects (15 males and 9 females; mean age = 18.5, range = 13–25 years). Four SRC patients and 17 control subjects participated in a previous cross-sectional study (35). Three control subjects were excluded due to excessive motion during neuroimaging assessment. Among control subjects that made up the normal control atlas, their past medical history was significant for remote concussion in 7/24 (29.2%) subjects. One SRC patient was on medication for a post-injury anxiety and sleep disorder during both the initial and follow-up neuroimaging. Additional clinical and neuroimaging characteristics of the SRC patients are summarized in Table 1.

**Table 1:** **Summary of demographic and clinical features of adolescent sports-related concussion patients**.

Subject	Age at injury/gender/sport	PMHx	LOC/PTA	Classification/co-existing conditions/predominant symptoms	Duration of symptoms at initial imaging (days)	Abnormal voxel counts (at initial imaging, *p* < 0.005)	Treatment	Clearance measures	Total duration of symptoms (days)	Abnormal voxel counts (at follow-up imaging, *p* < 0.005)
SRC 1	16 F, Soccer	Concussion (3), motion sensitivity	No/no	PCS/Vestibulo-ocular PCD No co-existing condition Balance, dizziness, visual problems, excessive sleep	279	Greater: 586	Vestibular physiotherapy	Clinical	329	Greater: 496
						Less: 170				Less: 502
						Total: 756				Total: 998
SRC 2	15 F, Hockey	Concussion (1), ADHD	No/no	PCS/post-injury psychiatric disorder Separation anxiety disorder, anxiety disorder NOS, insomnia Anxiety, fatigue, insomnia, headache	188	Greater: 1088	Methylphenidate, fluoxetine, trazodone	Clinical Graded aerobic treadmill testing fNT	376	Greater: 629
						Less: 0				Less: 99
						Total: 1088				Total: 728
SRC 3	16 F, Cycling	Concussion (1)	Yes/yes	Physiological PCD No co-existing condition Headache, difficulty focusing, sensitivity to light and sound, irritability	174	Greater: 15,250	Sub-maximal exercise therapy Cervical PT	Clinical	320	Greater: 1534
						Less: 49				Less: 1828
						Total: 15,299				Total: 3362
SRC 4	15 M, Football	Concussion (2), migraine	No/no	Physiological PCD No co-existing condition Headache, dizziness, light sensitivity	33	Greater: 8670	Sub-maximal exercise therapy	Clinical Graded aerobic treadmill testing fNT	213	Greater: 2653
						Less: 10				Less: 24
						Total: 8680				Total: 2677
SRC 5	15 M, Football	Concussion (1)	No/yes	Acute concussion BPPV Headache, nausea, difficulty remembering, postural imbalance, vertigo	13	Greater: 3876	Vestibular physiotherapy (BPPV)	Clinical Graded aerobic treadmill testing	41	Greater: 2709
						Less: 363				Less: 338
						Total: 4239				Total: 3047
SRC 6	17 M, Hockey	Concussion (1)	No/yes	Acute concussion No co-existing condition Headache, fatigue, sensitivity to light and sound	7	Greater: 846	Conservative management	Clinical Graded aerobic treadmill testing cNT	34	Greater: 64
						Less: 148				Less: 310
						Total: 994				Total: 374

*SRC, sports-related concussion patient; PMHx, past medical history; M, male; F, female; LOC, loss of consciousness; PTA, post-traumatic amnesia; PCS, post-concussion syndrome; PCD, post-concussion disorder; AHDH, attention deficit hyperactivity disorder; NOS, not otherwise specified; fNT, formal neuropsychological testing; cNT, computerized neurocognitive testing; PT, physiotherapy; BPPV, benign paroxysmal positional vertigo*.

### Study Tolerability

All subjects successfully completed the full imaging study. One SRC patient reported transient and self-limiting headache and fatigue during initial neuroimaging assessment.

### Structural Neuroimaging

Structural neuroimaging studies were normal in all subjects. No evidence of traumatic abnormalities was detected among SRC patients.

### End-Tidal Gas Targeting and Hemodynamics

No significant differences in baseline PETCO_2_ (41.6 ± 3.8 mmHg – first imaging period and 41.3 ± 3.7 mmHg – second imaging period; *p* = 0.822 paired *t*-test), and the change in CO_2_ (ΔCO_2_) during CO_2_ targeting (4.3 ± 0.7 mmHg first imaging, 4.2 ± 1.1 mmHg second imaging; *p* = 0.874) were detected. Thus, the patients were exposed to the same CO_2_ stimulus during BOLD imaging on both occasions.

### BOLD Cerebrovascular Responsiveness (First-Level Analysis)

There was no significant difference between the control subject atlas and SRC patient BOLD responses to CO_2_ (*p* = 0.001; 10 voxel cluster size). The mean BOLD response to the CO_2_ stimulus was 83 ± 12% in the SRC patients and 85 ± 6% in the control subjects (*p* = 0.668 between groups). The mean BOLD inverse response was 0.5 ± 0.4% in the SRC patients and 0.5 ± 0.4% in controls (*p* = 0.774 between groups). The number of voxels masked out by the inclusive mask [(gm + wm) − (B0_inhomogeneity + dilated_ventricles)] was 20,980 ± 3900 in the SRC group at imaging period 1 and 21,290 ± 4400 at imaging period 2, indicating no difference in number of voxels masked out between time periods (*p* = 0.919). The proportion of voxels masked out by the B0 field inhomogeneities represented 5.4 ± 1.7% of total voxel counts during imaging period 1 and 4.5 ± 1.7% in imaging period 2. Dilated ventricle masking represented 5.7 ± 3.2 and 6.9 ± 2.1%, respectively.

### BOLD Cerebrovascular Responsiveness (Second-Level Analysis)

**Figure 1:**
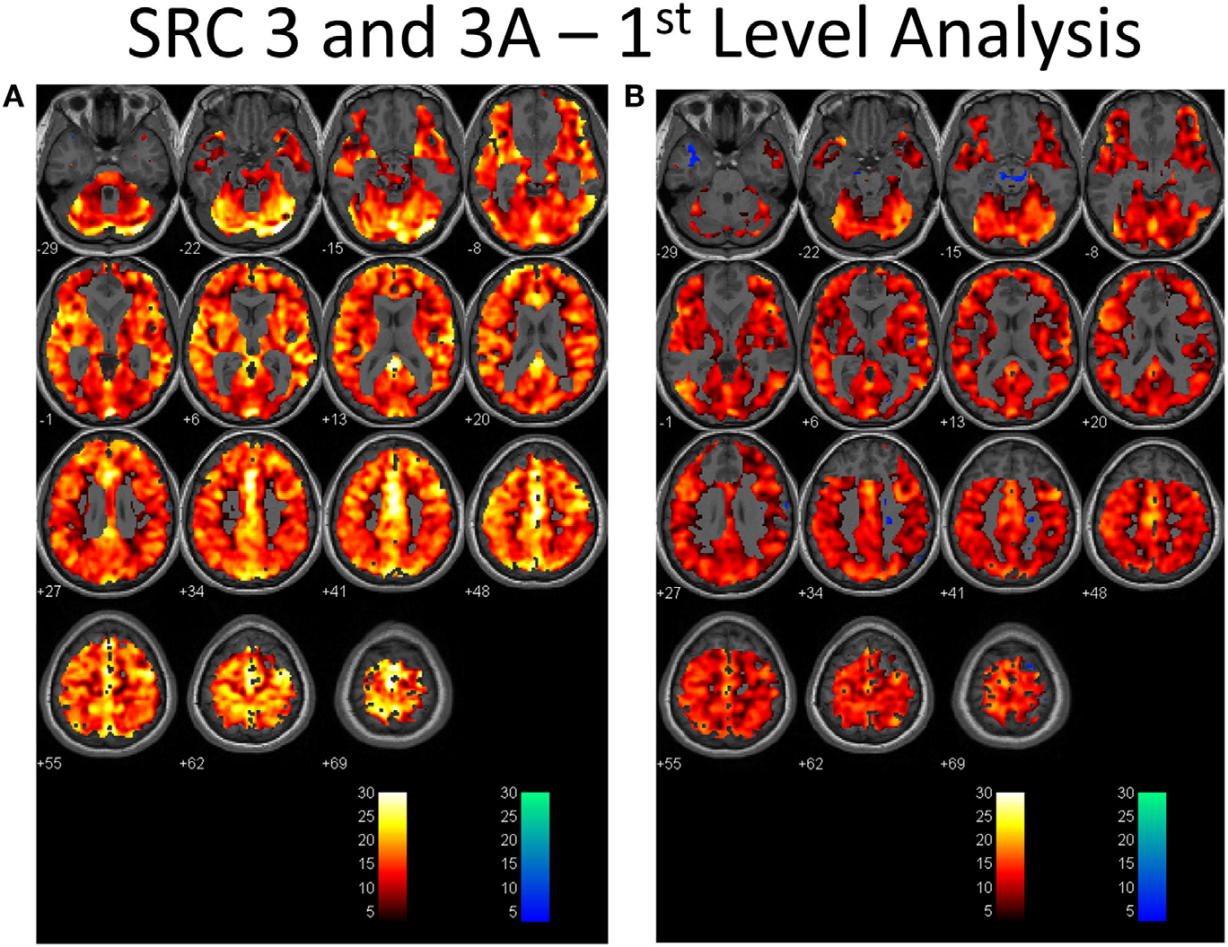
**First-level analysis of longitudinal brain MRI CO_2_ stress testing in one physiological post-concussion disorder patient during the symptomatic (A) and clinically recovered stages of injury (B)**. In each case the hot voxels (orangish hues) indicate where voxels have responded (at the *p* = 0.001 level; *t*-statistic = 3.11) to the triple CO_2_ stimulus as delivered by the model-based prospective end-tidal targeting device as outlined in the methodology based on the general linear model constructed in the SPM analysis. A colored voxel indicates that the BOLD response increased when the CO_2_ stimulus increased and decreased as the CO_2_ stimulus decreased during the applied triple stimulus. The cold voxels (bluish hues) indicate where voxels have responded (at the *p* = 0.001 level; *t*-statistic = 3.11) in an inverse manner to the triple CO_2_ stimulus – that is the BOLD signal decreased when the CO_2_ signal increased and vice versa. Where the voxels remain gray the anatomic image template shows through indicating that the chosen level of statistical significance was not attained. In each study, an individualized inclusive mask was applied to eliminate artifactual voxels (see description in the methodology and a representative mask in Supplementary Material). The color bar numbers indicate the value of the *t*-statistic attained for the color displayed.

**Figure 2:**
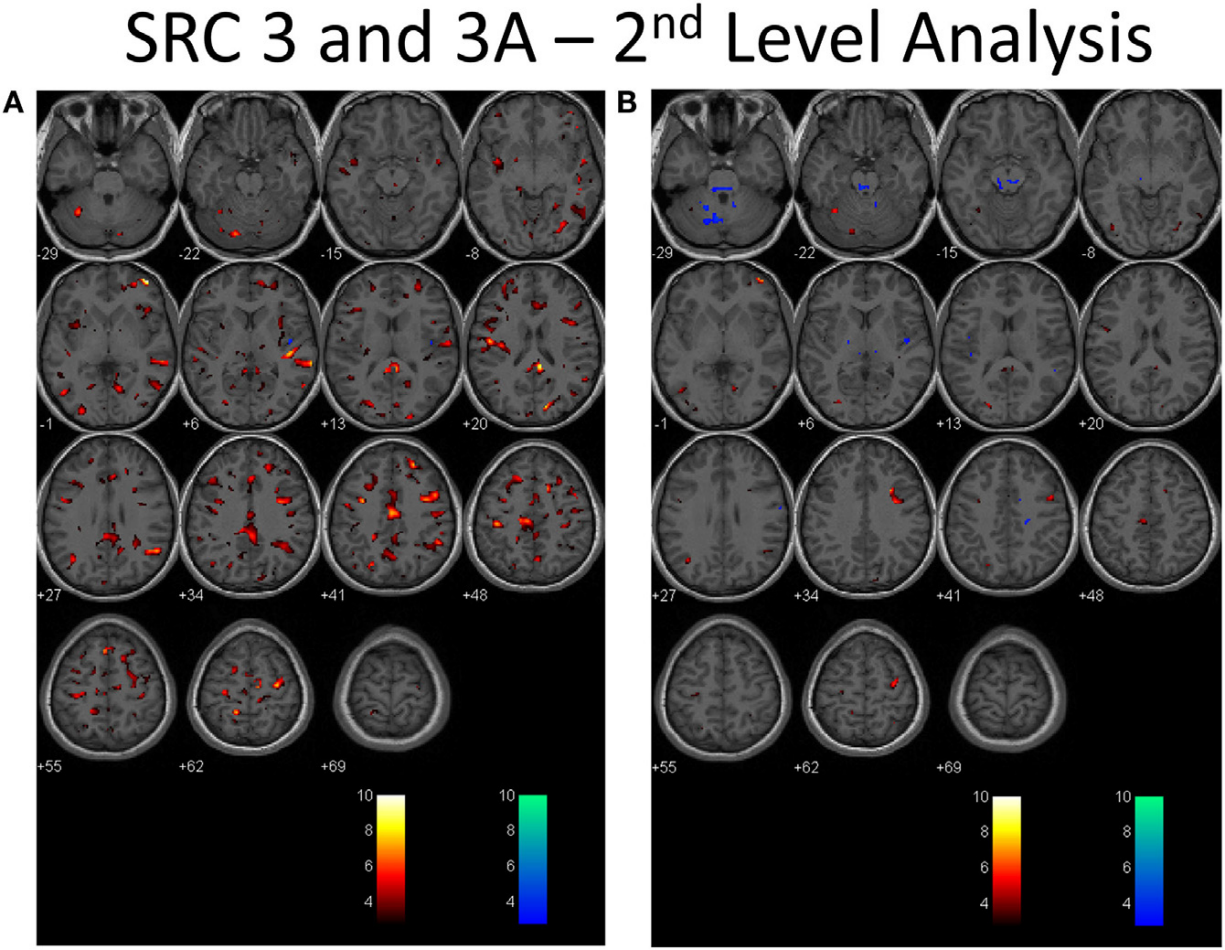
**Second-level analysis of longitudinal brain MRI CO_2_ stress testing in the same physiological post-concussion disorder patient illustrated in Figure 1 during the symptomatic (A) and clinically recovered stages of injury (B) compared with the stand-alone control atlas**. Results are reported at the *p* = 0.005 level for cut-off of significant voxels (*t*-statistic value = 2.82 in this case). Hot color voxels (orangish hues) indicate where BOLD CVR response is significantly greater than the mean value for the collated control group images and the cool color voxels (blue hues) indicate where the BOLD CVR response is significantly less when compared with the mean value for the control group. The scale indicates the color scheme as expressed in *t*-values. This approach permits quantitation of the longitudinal differences evident to the eye as seen in Figure 1 to be displayed.

Second-level analysis comparing individual SRC patients to the healthy control atlas at their initial neuroimaging tests, during the symptomatic phase of injury, demonstrated patient-specific alterations in CVR in all patients with a predominant pattern of increased CVR. Follow-up neuroimaging, during the clinically recovered stage of injury, demonstrated relatively stable CVR in two patients, and improved but persistently abnormal CVR in four patients. Examples of longitudinal CVR assessment using brain MRI CO_2_ stress testing is shown in Figures 1 and 2. Images indicate voxel counts at the *p* = 0.005 level. The full complement of patient study images is available in Supplementary Material.

## Discussion

In this pilot study, when assessed for abnormality by comparison to a normal control atlas, patient-specific qualitative and quantitative alterations in CVR were detected in all SRC patients; with the highest abnormal voxel counts observed among patients with acute concussion and physiological post-concussion disorder. When assessed for changes longitudinally, two patients with physiological post-concussion disorder (SRC 3 and 4) demonstrated improvements in CVR that nevertheless remained abnormal. Two other PCS patients with more chronic symptoms, one with isolated vestibulo-ocular dysfunction (SRC 1) and one who developed a post-injury psychiatric disorder (SRC 2), demonstrated relatively stable CVR measures with little change over time. For the two acute SRC patients (SRC 5 and 6), both demonstrated improvements in CVR that nevertheless remained persistently abnormal despite clinical recovery.

The results of this pilot study, therefore, provide confirmatory evidence that SRC patients display both qualitative and quantitative patient-specific alterations in CVR that can be detected by brain MRI CO_2_ stress testing. Our findings also suggest that alterations in CVR may account for some of the clinical findings in patients with acute concussion and physiological post-concussion disorder, but that CVR changes may play a less prominent role in those with isolated vestibulo-ocular dysfunction and post-injury psychiatric disorders. Our results are in agreement with previous work that demonstrated alterations in mean resting CBF in groups of pediatric and collegiate SRC patients that persisted beyond clinical and neurocognitive recovery (18, 23). Whether alterations in resting CBF and CVR that persist beyond clinical recovery place these athletes at an elevated risk of future injury, or lead to long-term effects of cumulative brain injury, requires further study. It is possible that the vessels marked by abnormal CVR may, like other tissues, be more vulnerable to re-injury, or worsened physiological dysfunction, with repeated head trauma. The persistence of CVR abnormalities detected months after injury in many of these patients indicates that much longer evaluations are required to document the natural history of cerebrovascular dysfunction following concussion. Our preliminary experience with this population suggests that concussion may be characterized by a predominant pattern of increased CVR during the acute or sub-acute phase and a predominantly reduced pattern of CVR in the chronic phase. If CVR is an adequate biomarker for cerebrovascular dysfunction following concussion, additional study will be required to further refine the criteria for abnormal CVR and the adequacy of neurovascular recovery as well as take into account potential artifact related to data analysis and fluctuations in CVR over time among normal subjects.

Brain MRI CO_2_ stress testing meets several important requirements for a neuroimaging assessment tool that can provide longitudinal CVR assessments in concussion patients. First, as demonstrated here and in previous studies (34, 35) this test is safe and well tolerated among acute SRC and PCS patients and does not require exposure to radiation, provocative breathing maneuvers, or intravenous agents. Second, this technique delivers a precise, quantifiable, and reproducible CO_2_ stimulus under isoxic conditions, thereby allowing a consistent vasoactive stimulus to be applied during serial assessments without the confounding effects of alterations in PETO_2_ and individual variability in respiratory physiology (32, 38). Third, this technique generates qualitative and quantitative biomarkers that can be compared with other clinical outcomes in individual SRC patients (33). Although there were subtle 1–2 mmHg differences in the CO_2_ stimulus applied across subjects and time-points, this level of end-tidal gas control is similar to that achieved with mechanical ventilation in other CVR studies (39, 40), and represents a level of precision and reproducibility that is unachievable with other vasoactive stimuli including breath-holding, acetazolamide, and inhaled CO_2_ such as carbogen (32, 33).

This pilot study has several important limitations. First, the number of SRC patients who underwent longitudinal CVR assessment is small. Patients were recruited from a tertiary pediatric concussion program that may have selected for more severely injured SRC patients. Second, objective measures of clinical concussion recovery, including graded aerobic treadmill testing and formal neuropsychological testing, were not used to confirm physiological and neurocognitive recovery in all patients, and patients were not imaged at uniform and pre-selected time periods. Some patients had returned to contact and collision sports prior to follow-up neuroimaging assessment in which cases the effects of unreported concussions or sub-concussion injuries may have affected follow-up imaging results. Third, masking of the BOLD EPI for the second-level analysis removed from consideration some of the prefrontal and frontal cortex and periventricular white matter in each case that may have resulted in under-detection of abnormal voxels. Lastly, although previous work has demonstrated excellent within-day reliability of CVR assessment in pediatric subjects using this technique (41), longitudinal CVR assessments were not performed among the healthy control subjects. This limitation should be considered in future studies.

In conclusion, this study provides confirmatory empirical evidence that SRC is associated with patient-specific alterations in CVR. Larger prospective studies are needed to confirm these preliminary findings and examine the utility of brain MRI CO_2_ stress testing as a clinical tool to help guide the evaluation, classification, prognostication, and longitudinal management of SRC patients.

## Author Contributions

WM – experimentation, data collation, analysis, interpretation, and writing. MJE – experimentation, data collation, analysis, interpretation, and writing. LR – experimentation, data collation, analysis, interpretation, and writing. MM – experimentation and data collation. PP – experimentation and data collation. BD – analysis and statistical consultation. ME – analysis and interpretation. DM – analysis, interpretation, and writing. JD – analysis, interpretation, and writing. JF – analysis, interpretation, and writing.

## Conflict of Interest Statement

JF, JD, and DM are senior scientists at Thornhill Research Inc., (TRI), a company affiliated with the University Health Network that developed the RespirAct™, a non-commercial research tool assembled by TRI to enable cerebrovascular reactivity studies. The remaining authors declare that the research was conducted in the absence of any commercial or financial relationships that could be construed as a potential conflict of interest.
