# Supplementary figures and images for: Longitudinal Brain Magnetic Resonance Imaging CO2 Stress Testing in Individual Adolescent Sports-Related Concussion Patients: A Pilot Study
Source: Front Neurol. 2016 Jul 8;7:107. doi: 10.3389/fneur.2016.00107 (PMC4937024; doi:10.3389/fneur.2016.00107)

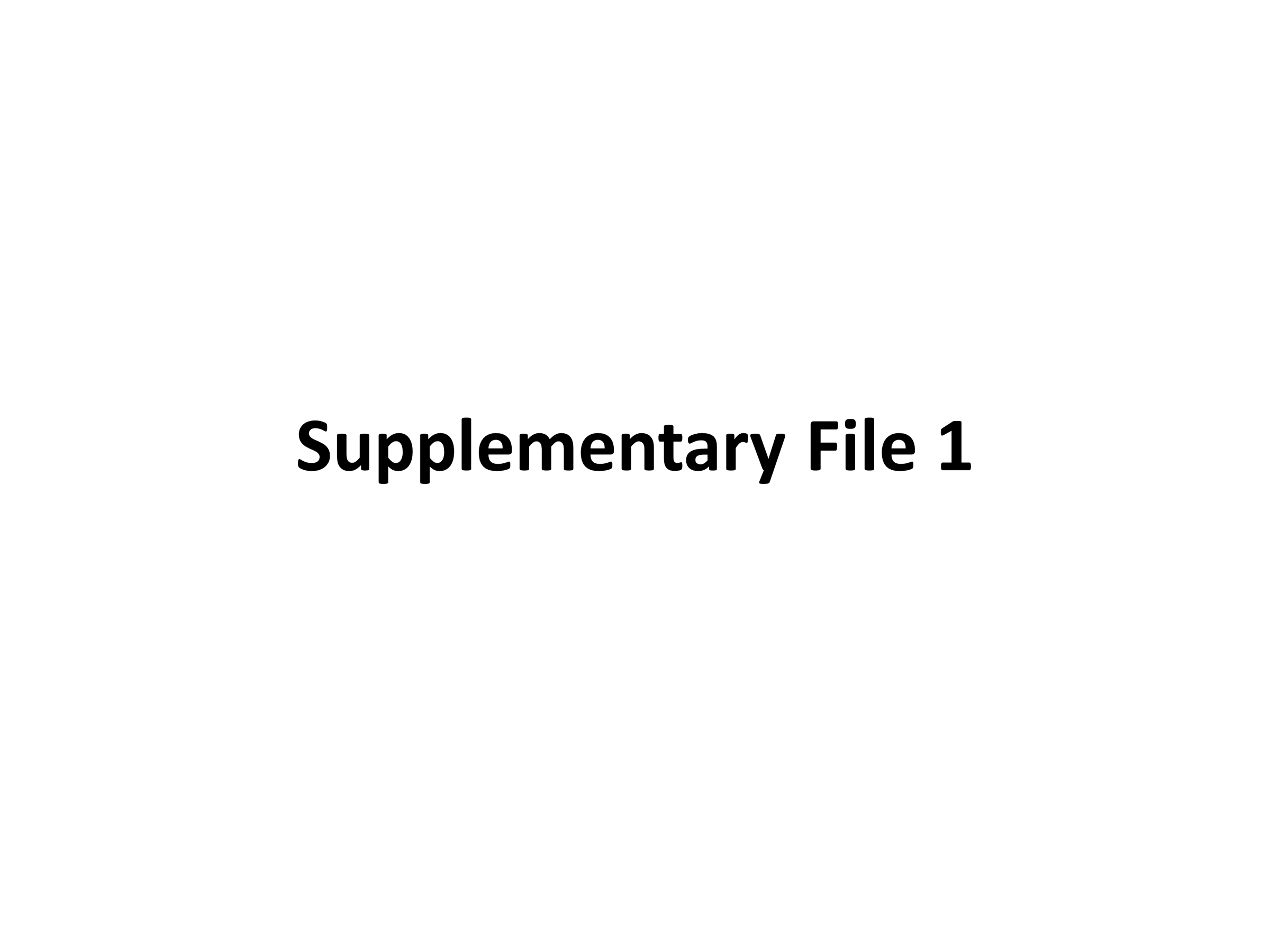

Supplement: Supplementary file 1 [file presentation_1.zip › AD_Long_Supplementary File 1/Slide1.TIF]

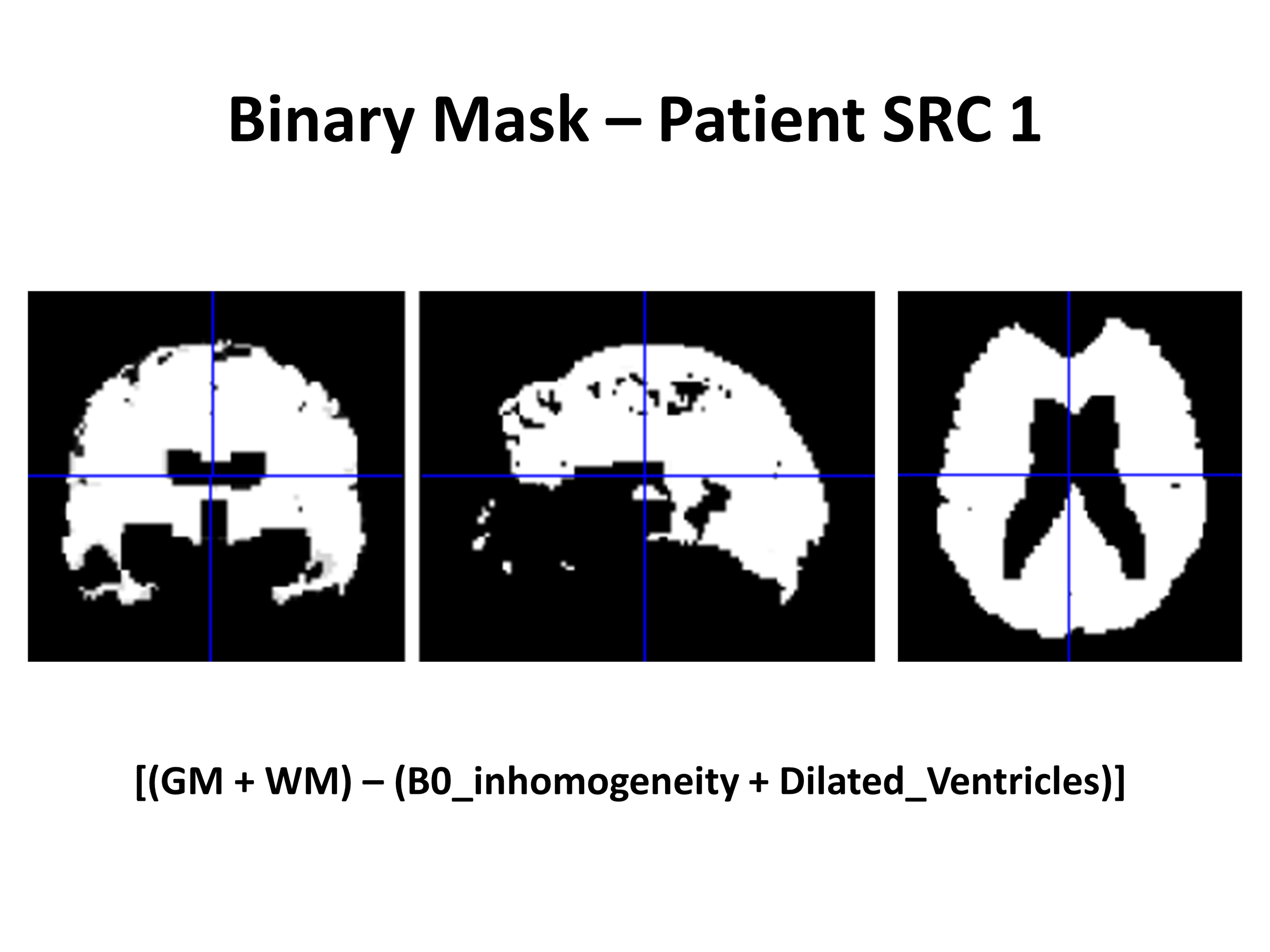

Supplement: Supplementary file 1 [file presentation_1.zip › AD_Long_Supplementary File 1/Slide2.TIF]

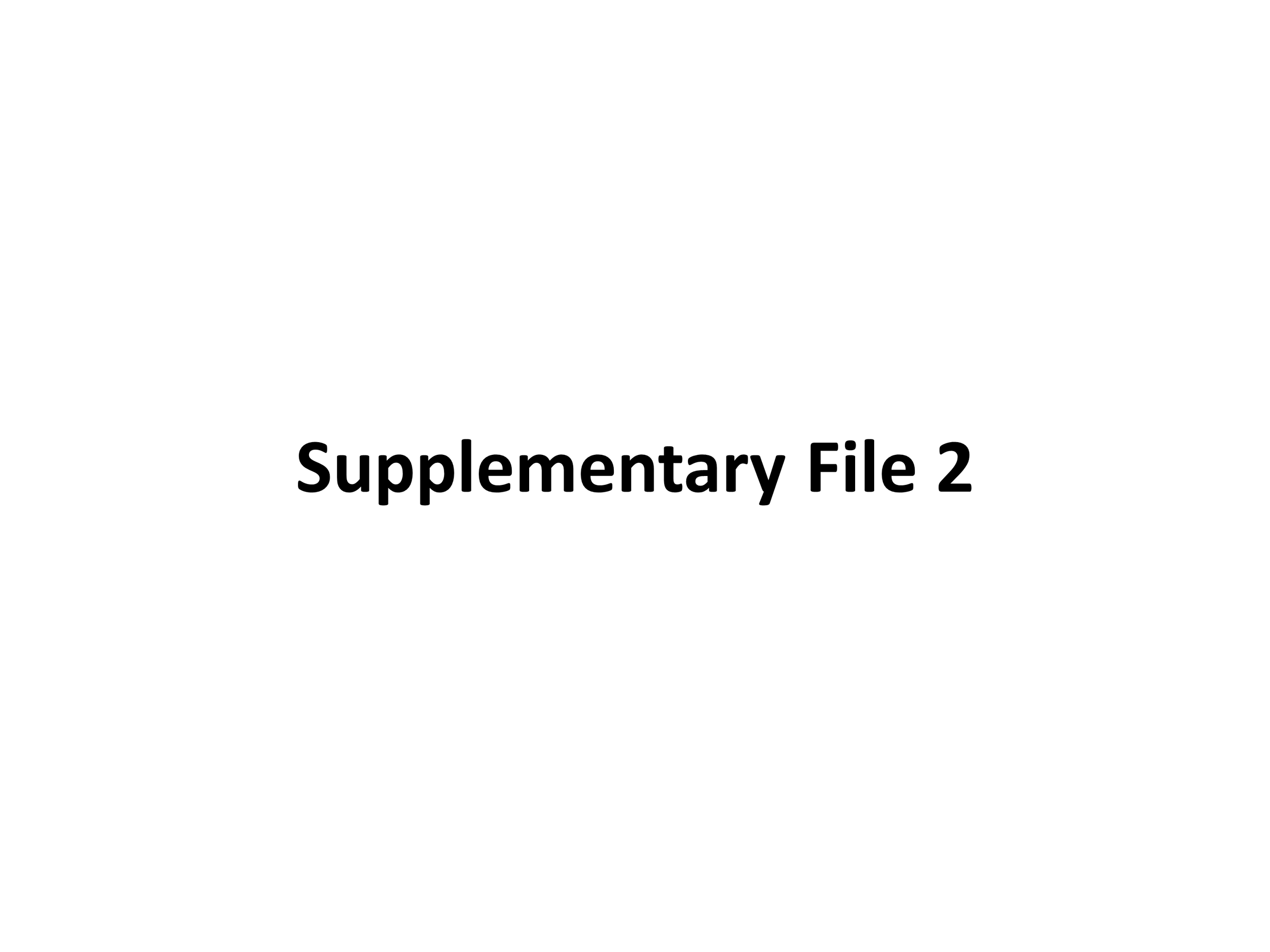

Supplement: Supplementary file 2 [file presentation_2.zip › AD_Long_Supplementary_File_2/Slide1.TIF]

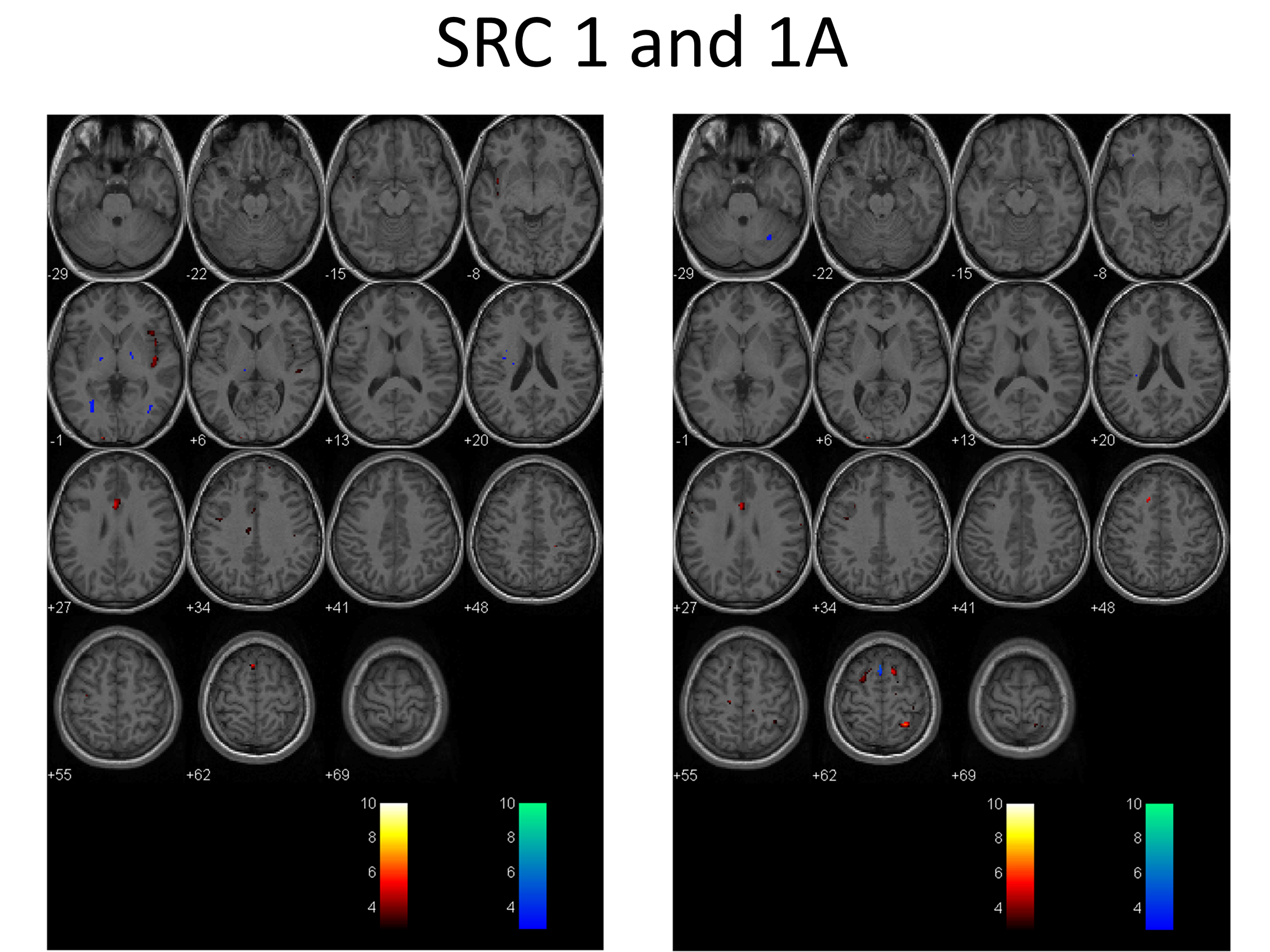

Supplement: Supplementary file 2 [file presentation_2.zip › AD_Long_Supplementary_File_2/Slide2.TIF]

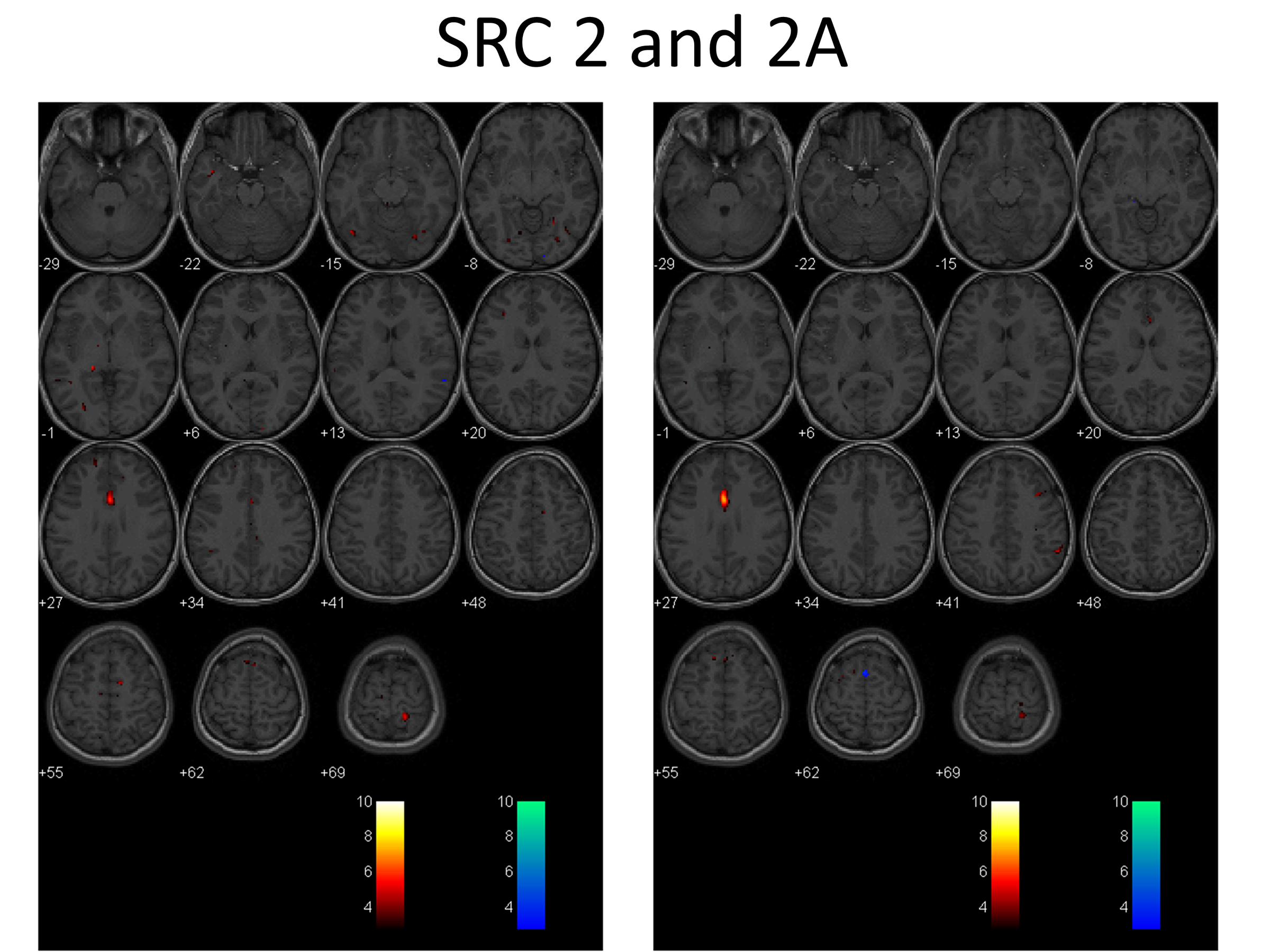

Supplement: Supplementary file 2 [file presentation_2.zip › AD_Long_Supplementary_File_2/Slide3.TIF]

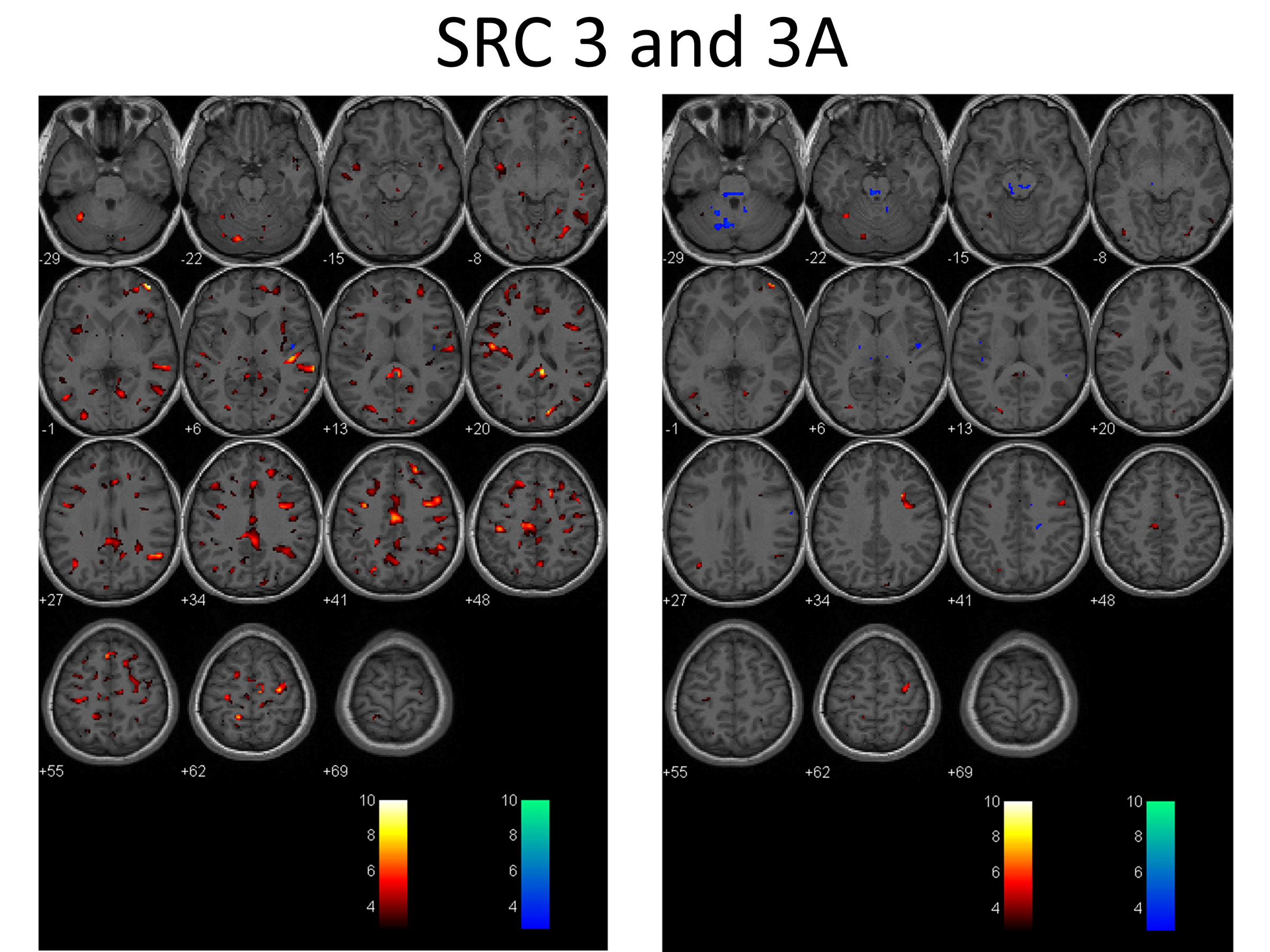

Supplement: Supplementary file 2 [file presentation_2.zip › AD_Long_Supplementary_File_2/Slide4.TIF]

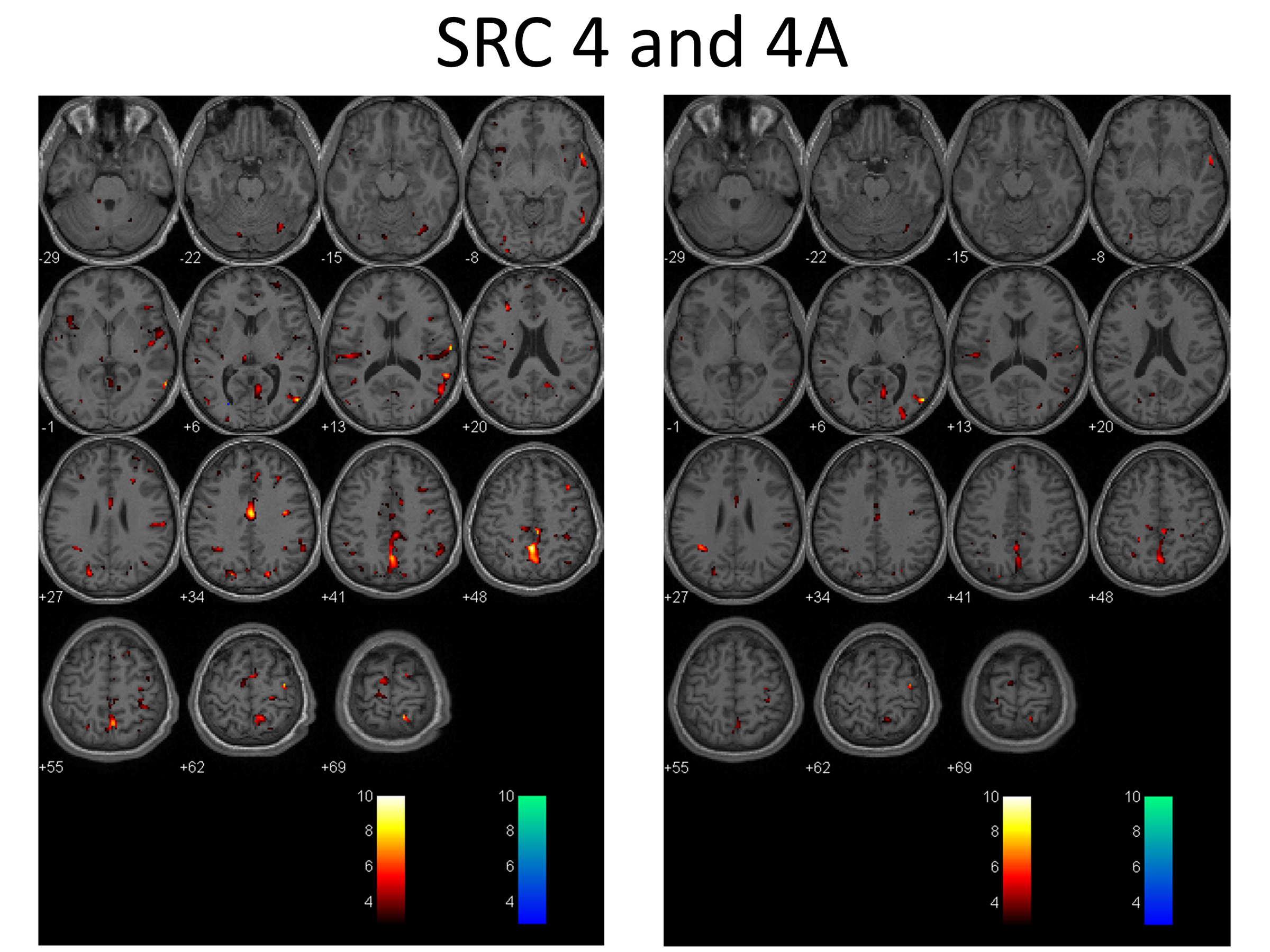

Supplement: Supplementary file 2 [file presentation_2.zip › AD_Long_Supplementary_File_2/Slide5.TIF]

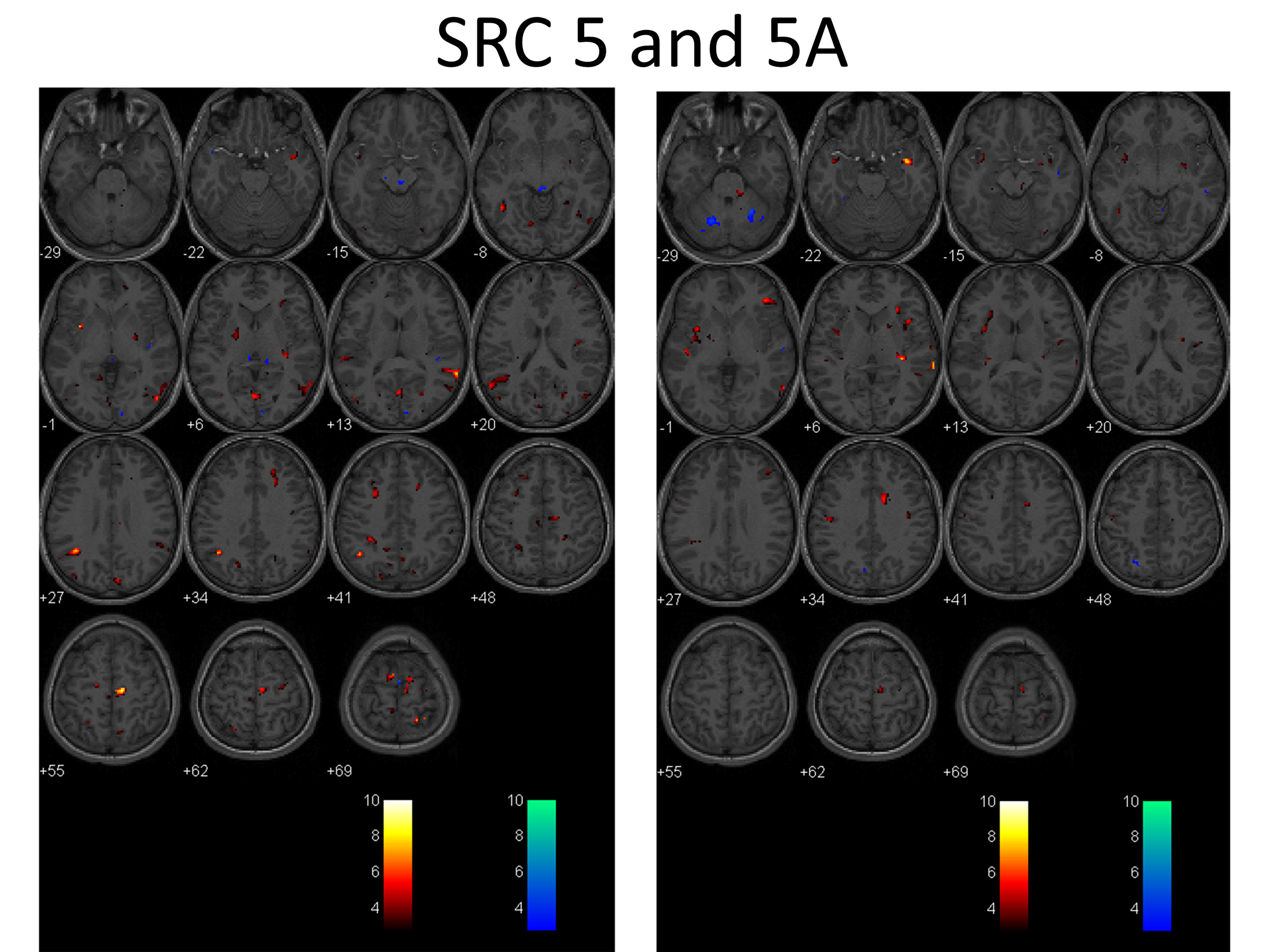

Supplement: Supplementary file 2 [file presentation_2.zip › AD_Long_Supplementary_File_2/Slide6.TIF]

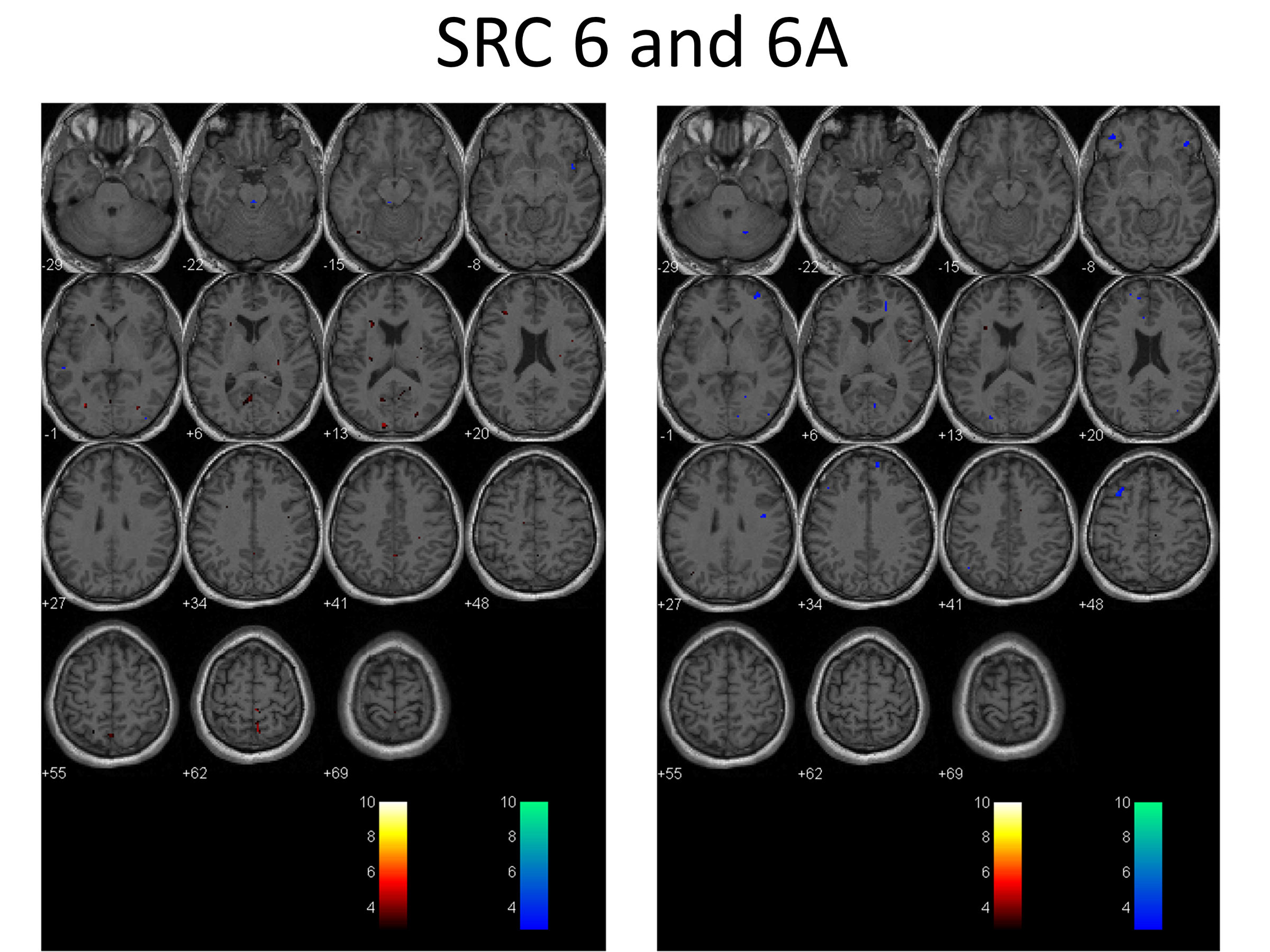

Supplement: Supplementary file 2 [file presentation_2.zip › AD_Long_Supplementary_File_2/Slide7.TIF]
